# Multiple neuropeptides produced by sex-specific neurons control activity of the male accessory glands and gonoducts in the silkworm *Bombyx mori*

**DOI:** 10.1038/s41598-019-38761-x

**Published:** 2019-02-19

**Authors:** Daniel Čižmár, Ladislav Roller, Miriam Pillerová, Karel Sláma, Dušan Žitňan

**Affiliations:** 1 Institute of Zoology, Slovak Academy of Sciences, Dúbravská cesta 9, 84506 Bratislava, Slovakia; 2 Biology Centre of Czech Academy of Sciences, Institute of Entomology, Drnovská 507, 16100 Praha 6, Czech Republic

## Abstract

The male accessory glands (AG) and gonoducts of moths develop during metamorphosis and are essential for successful fertilization of females. We found that these reproductive organs are innervated by a sex-specific cluster of peptidergic neurons in the posterior 9^th^ neuromere of the terminal abdominal ganglion (TAG). This cluster of ~20 neurons differentiate during metamorphosis to innervate the accessory glands and sperm ducts. Using immunohistochemistry and *in situ* hybridization (ISH) we showed that these neurons express four neuropeptide precursors encoding calcitonin-like diuretic hormone (CT-DH), allatotropin (AT) and AT-like peptides (ATLI-III), allatostatin C (AST-C), and myoinhibitory peptides (MIPs). We used contraction bioassay *in vitro* to determine roles of these neuropeptides in the gonoduct and accessory gland activity. Spontaneous contractions of the seminal vesicle and AG were stimulated in a dose depended manner by CT-DH and AT, whereas AST-C and MIP elicited dose dependent inhibition. Using quantitative RT-PCR we confirmed expression of receptors for these neuropeptides in organs innervated by the male specific cluster of neurons. Our results suggest a role of these neuropeptides in regulation of seminal fluid movements during copulation.

## Introduction

Insects are the most widespread and common group of terrestrial animals due to their effective reproductive strategies. High speed of reproduction is also very important attribute of all economically important crop pests. Therefore, the understanding of regulatory mechanisms required for successful reproduction has always been an important issue of basic and applied research.

In the silkmoth *Bombyx mori* the reproductive system consists of paired gonads (testes or ovaries), accessory glands and gonoducts. The male gonoducts are composed of the vasa deferentia, seminal vesicles and ejaculatory duct. The accessory glands are also joined to the seminal vesicles^1,2^. These tubular glands produce a wide variety of bioactive compounds facilitating sperm transfer and peptides influencing behavior of the female after mating^3^. Movements of seminal fluids within the reproductive organs are facilitated by the visceral muscles that form outer layer of gonoducts and associated glands. This musculature is innervated by neurons from the terminal abdominal ganglion (TAG) which have been first described in the tobacco moth *Manduca sexta*^4^. Using cobalt backfilling, several clusters of sex-specific neurons (named imaginal midline neurons, IMNs) have been identified in the TAG^5^. IMNs differentiate through early pupal stages and their number is multiplied during metamorphosis in the anterior clusters in females and in the posterior cluster in males. In males a posterior cluster of IMN exit via terminal nerves to innervate the sperm duct and accessory glands^6,7^.

Like many other biological processes, reproduction is controlled by neuropeptides. Approximately 40 neuropeptide families have been identified in *B. mori*^8–13^. Using *in situ* hybridization (ISH) and immunohistochemistry we identified sex-specific differences in expression of several neuropeptide genes in the TAG during comprehensive mapping of neuropeptide localization in the central nervous system (CNS) of *B. mori*^7,8^. Here, we describe developmental changes in expression of calcitonin-like diuretic hormone (CT-DH), allatotropin (AT), allatostatin C (AST-C) and myoinhibitory peptide (MIP) in the TAG. These different neuropeptides are coexpressed in a male-specific cluster of neurons that differentiates during metamorphosis and innervates the male reproductive organs. We also detected expression of receptors for these neuropeptides in the male gonoducts and accessory glands. These data indicate a complex neuropeptide-receptor signaling that controls activity of the male reproductive organs during mating. Therefore, possible roles of these neuropeptides on spontaneous muscle activity of the accessory glands, seminal vesicles and ejaculatory duct were examined *in vitro* using electrophysiology.

## Results

### Expression of neuropeptides in sex-specific neurons of the posterior TAG

**Figure 1:**
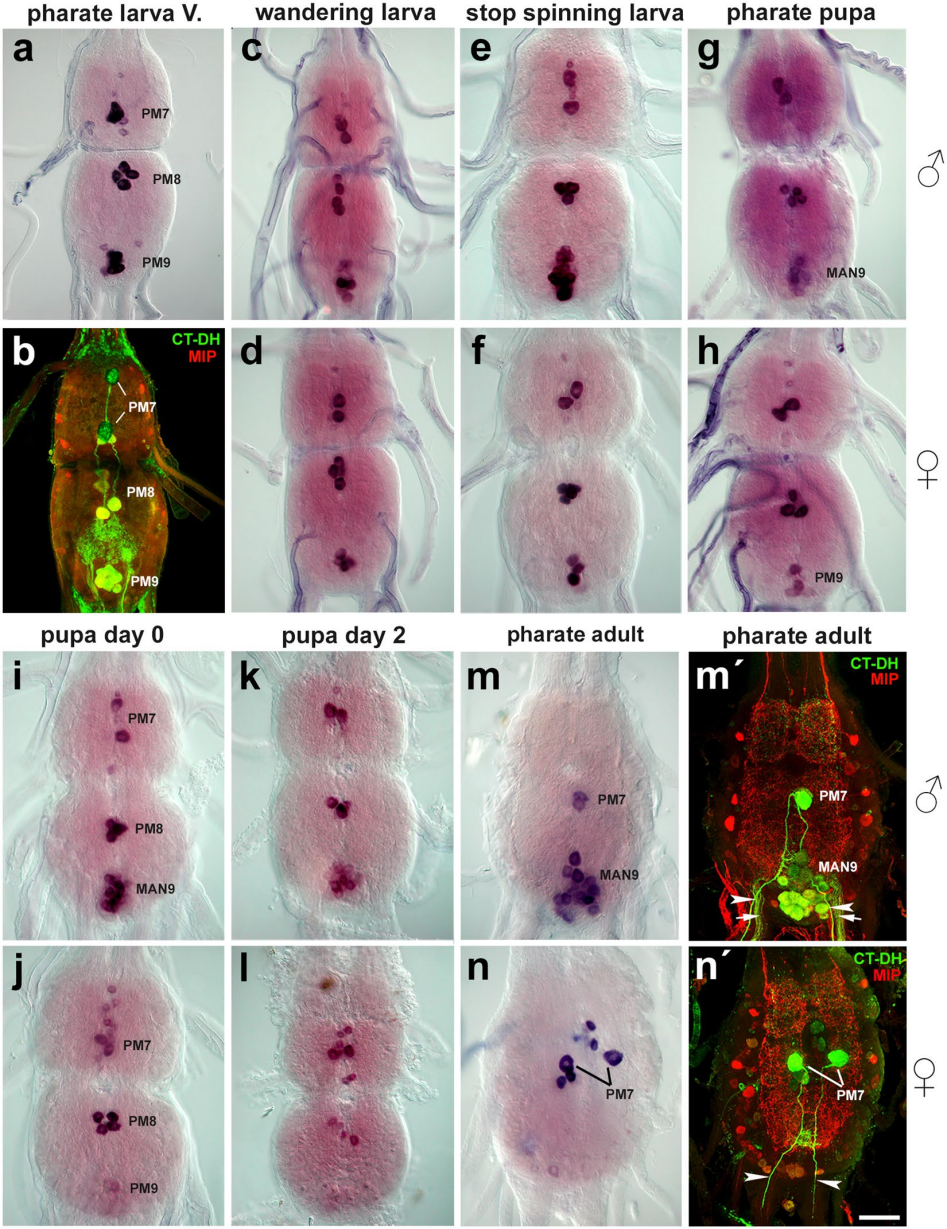
Sex-specific differences in expression of CT-DH in the TAG during metamorphosis. (**a**–**h**) ISH and immunohistochemical staining revealed restricted CT-DH expression in prominent midline neurons (PM7, PM8 and PM9) and a few smaller cells in larvae and pharate pupae of both sexes. (**e**–**n**) Male-specific adult neurons (MAN9) started to differentiate in spinning 5th instar larvae and their number increased to ~20 in pharate adults, while female TAG showed considerably reduced expression of CT-DH in AN9 after pupation (**j,l,n**). Note that PM7 showed CT-DH expression throughout the metamorphosis and project axons into the terminal nerves (**b,m′,n′**; arrowheads), whereas PM8 disappear in pharate adults of both sexes (**m,n**). Colocalization of CT-DH (green) and MIP (red) was detected in PM8, PM9 and MAN9 (**b,m′**; yellow) projecting into the terminal nerves (arrows). Scale bar = 50 μm.

**Figure 2:**
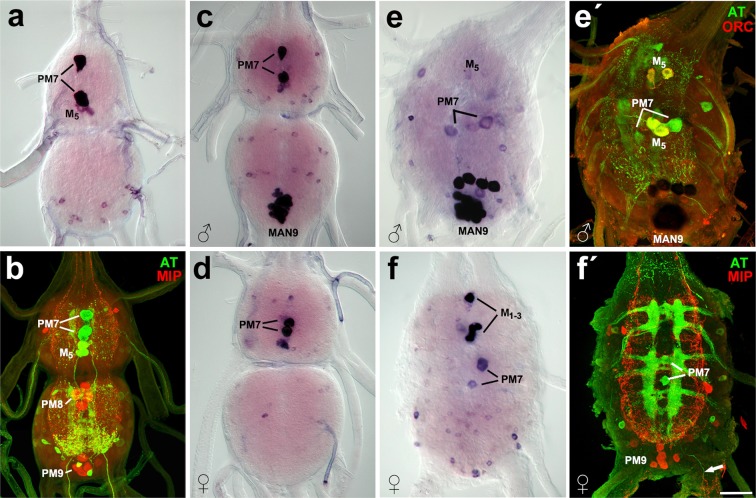
Developmental changes of allatotropin expression in the TAG. (**a,b**) In pharate larva strong AT expression was detected by ISH (**a**) or immunostaining (**b**) in midline neurons PM7 and medial neurosecretory M_5_, and several smaller cells in AN9. (**c**–**f**) Male-specific MAN9 neurons appeared in pharate pupae (**c**) and increased in size and number in pharate adults (**e,e′**). Note colocalization of AT-IR (green) and ORC-IR (red) in M_5_ cells (**e′**). Females showed AT expression in PM7, medial neurosecretory M_1–3,5_ (nomenclature according to Davis *et al*.^42^) and numerous small unidentified neurons (**d,f,f′**). Note AT-IR (green) in PM7 projecting into terminal nerves (arrow) and MIP-IR (red) in PM9-like neurons (**f′**). Scale bar = 50 μm.

**Figure 3:**
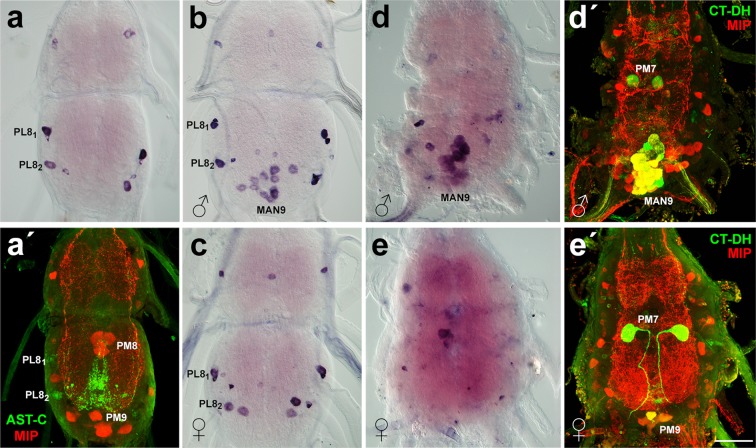
Sex-specific expression of allatostatin C in the TAG. (**a,a′**) Pharate larvae showed strong AST-C expression in proctodeal lateral neurons (PL8_1_ and PL8_2_) and a few small neurons in both males and females. Following immunostaining with antibodies to AST-C (green) and MIP (red) (**a′**) confirmed presence of AST-C in PL8_1_ and PL8_2_. (**b,c**) AST-C expression appeared in 10–12 MAN9 cells of male pharate pupae (**b**), but not in females (**c**). (**d**,**d′**) In pharate adults the number of MAN9 neurons expressing AST-C increased to 18–20. Identity of these cells detected with AST-C probe by ISH was confirmed by immunostaining with antibodies to CT-DH (green) and MIP (red) (**d′**). AST-C expression was absent in midline neurons of the female (**e**), however PM7 and PM9-like cells were detected in the same TAG with antibodies to CT-DH and MIP (**e′**). Note that PL8_1,2_ neurons disappeared in pharate adults (**d,e**). Scale bar = 50 μm.

**Figure 4:**
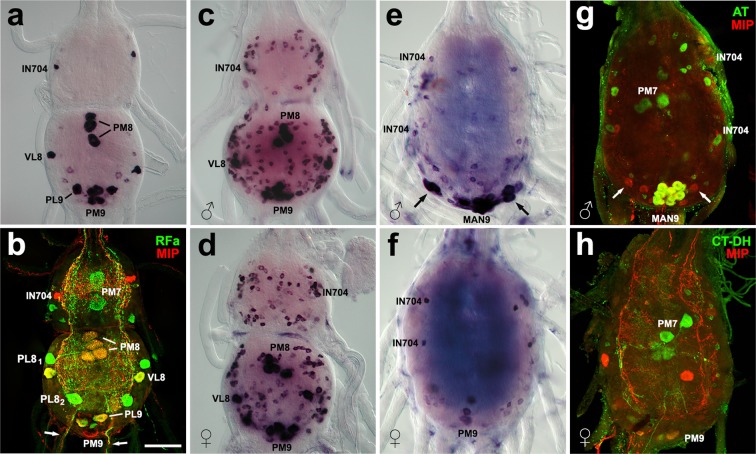
Developmental changes of MIP expression in the TAG. (**a,b**) ISH (**a**) and immunohistochemistry (**b**) in pharate larvae revealed strong MIP expression in lateral interneurons 704 (IN704) and neurons VL8, medio-lateral neurons PL9, and midline neurons PM8 and PM9 projecting axons to terminal nerves (arrows). (**b**) Double staining revealed colocalization of MIP- and FMRFamide-like IR in PM8, VL8 and PL9 neurons, but PM7 and PL8_1,2_ were only stained with FMRFamide antibody. (**c,d**) Additional 120–140 small neurons were detected in both sexes of pharate pupae, but no obvious MIP expression was observed in MAN9. (**e**–**h**) Sex-specific differences were apparent in pharate adults. (**e,g**) Strong MIP expression was detected by ISH (**e**) or immunostaining (**g**) in a cluster of MAN9 and additional neurons located more laterally (arrows). Colocalization of AT-IR (green) and MIP-IR (red) confirmed identity of MAN9 cells (yellow). (**f,h**) In females only 2–4 PM9-like neurons were detected by ISH (**f**) or double staining (**h**; yellow) with antibodies to CT-DH (green) and MIP (red). Scale bar = 50 μm.

Detailed analysis of neuropeptide expression revealed male-specific cluster of peptidergic neurons that differentiate during metamorphosis in the TAG. Using a combination of ISH and immunohistochemistry^7,14,15^, we detected expression of four different groups of neuropeptides (CT-DH, AT, ATLI-III, AST-C, and MIPs) in these neurons. This cluster contains ~20 posterio-medial neurons (~25 μm in diameter) in the male abdominal neuromere 9 (AN9) and we named them the Male Adult Neurons of AN9 (MAN9) (Figs 1–4).

### Calcitonin-like diuretic hormone

Both males and females of 5^th^ instar larvae show CT-DH expression in three groups of prominent midline neurons in the TAG which is composed of fused abdominal neuromeres 7–9 (AN7-9); a pair of large neurons in the AN7 (∼35 μm in diameter), four large neurons in the AN8 (∼30 μm) and 4–6 smaller neurons in the AN9 (∼25 μm) (Fig. 1a,b). These neurons project their axons via terminal nerves to innervate the hindgut and therefore we named them as proctodeal median neurons of AN7-9 (PM7-9). Reorganization of the CNS during metamorphosis (abdominal ganglion 6 was fused with TAG) was associated with considerable changes in CT-DH expression. PM8 neurons disappeared in both sexes several days after pupation, while the male-specific MAN9 differentiated in the vicinity of PM9 neurons (Fig. 1c–n). About 8–12 MAN9 expressed CT-DH in spinning and pharate pupae 1–3 days prior to ecdysis (Fig. 1c,e,g). The number of these neurons subsequently increased after pupation to 20 in pharate adult males (Fig. 1i,k,m). MAN9 were missing in females and only 2–4 PM9 show weak CT-DH expression in pupal and adult stages (Fig. 1j,l,n). Following ISH, antibodies to CT-DH and MIP were used for double staining to follow axonal projections of the TAG neurons (Fig. 1m′,n′). This approach revealed that MAN9 innervate the male spermiducts and accessory glands (see below).

### Allatotropin

The allatotropin gene is transcribed in three variants of alternatively spliced mRNA precursors. All three precursors encode allatotropin (AT) and various combinations of allatotropin-like peptides (ATLI-III)^7,16^. Expression of all three transcripts has recently been described in the entire CNS during development of *B. mori*^7^, so in this paper we will focus on the TAG. Larvae showed AT expression in two dorso-medial PM7 neurons and M_5_ neurosecretory cells in the AN7 and several small neurons in the AN9 (Fig. 2a,b). In pharate pupae of males, strong AT expression appeared in MAN9 (8–12 cells), but no neurons were detected in the posterior TAG of females (Fig. 2c,d). In pharate adult males, the number of MAN9 increased to 16–20 neurons. ISH with AT probe and following immunostaining with antibodies to AT, CT-DH and MIP showed an overlap in MAN9 cells and confirmed their identity (Fig. 2e,e′). In addition, colocalization of AT- and orcokinin-immunoreactivity (ORC-IR) was detected in M_5_ cells (Fig. 2e′). In female pharate adults, the AT probe and AT antibody reacted with numerous neurons, but no homologs of MAN9 cells were detected in the posterior region of the TAG (Fig. 2f,f′).

### Allatostatin C

Using ISH with AST-C probe and immunohistochemistry with AST-C antibody we detected two pairs of prominent neurons (∼25 μm in diameter) in AN8 of larvae and pharate pupae (Fig. 3a–c). These cells were identified as proctodeal lateral neurons 1 and 2 (PL8_1,2_) based on previous work^17^. Additional smaller lateral neurons were observed in AN7-8. Most these neurons vanished after pupation (Fig. 3d,e), but male-specific AST-C expression appeared in 8–10 MAN9 cells in pharate pupae and their number increased to ~20 in pharate adults. As described above, these neurons were never observed in females (Fig. 3b–e). Double staining with CT and MIP antibodies confirmed colocalization of multiple different neuropeptides in a cluster of MAN9 neurons in males and their absence in females (Fig. 3d′,e′)

### Myoinhibitory peptides

**Figure 5:**
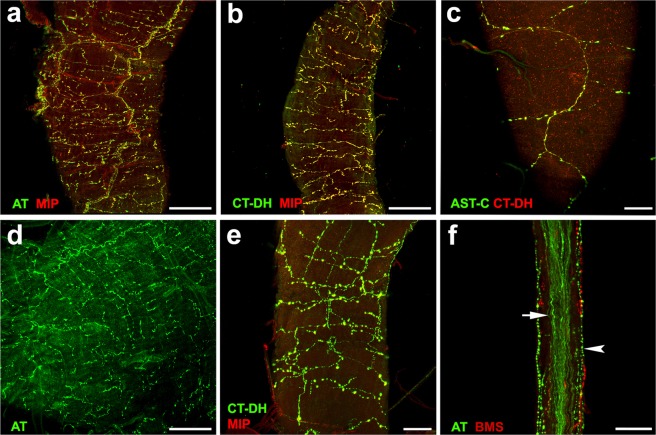
Innervation of the male reproductive system by MAN9. (**a,b**) Colocalization of AT-IR, CT-DH-IR (green) and MIP-IR (red) in axons innervating the accessory glands (yellow). (**c**) CT-DH-IR (red) and AST-C-IR (green) was also colocalized in innervation of basal part of the vas deferens (yellow). (**d**) A dense network of axonal branches and terminals with varicosities stained with AT antibody on surface of the seminal vesicle. (**e**) Antibodies to CT-DH and MIP revealed axonal projections containing varicosities on surface of the ejaculatory duct. (**f**) A bundle of MAN9 axons within the terminal nerve stained with AT antibody (arrow). Fine axonal processes with varicosities on surface of the terminal nerve (arrowhead) originate from different neurons producing AT and BMS. Scale bars a,b,d = 100 μm, c,e,f = 50 μm.

**Figure 6:**
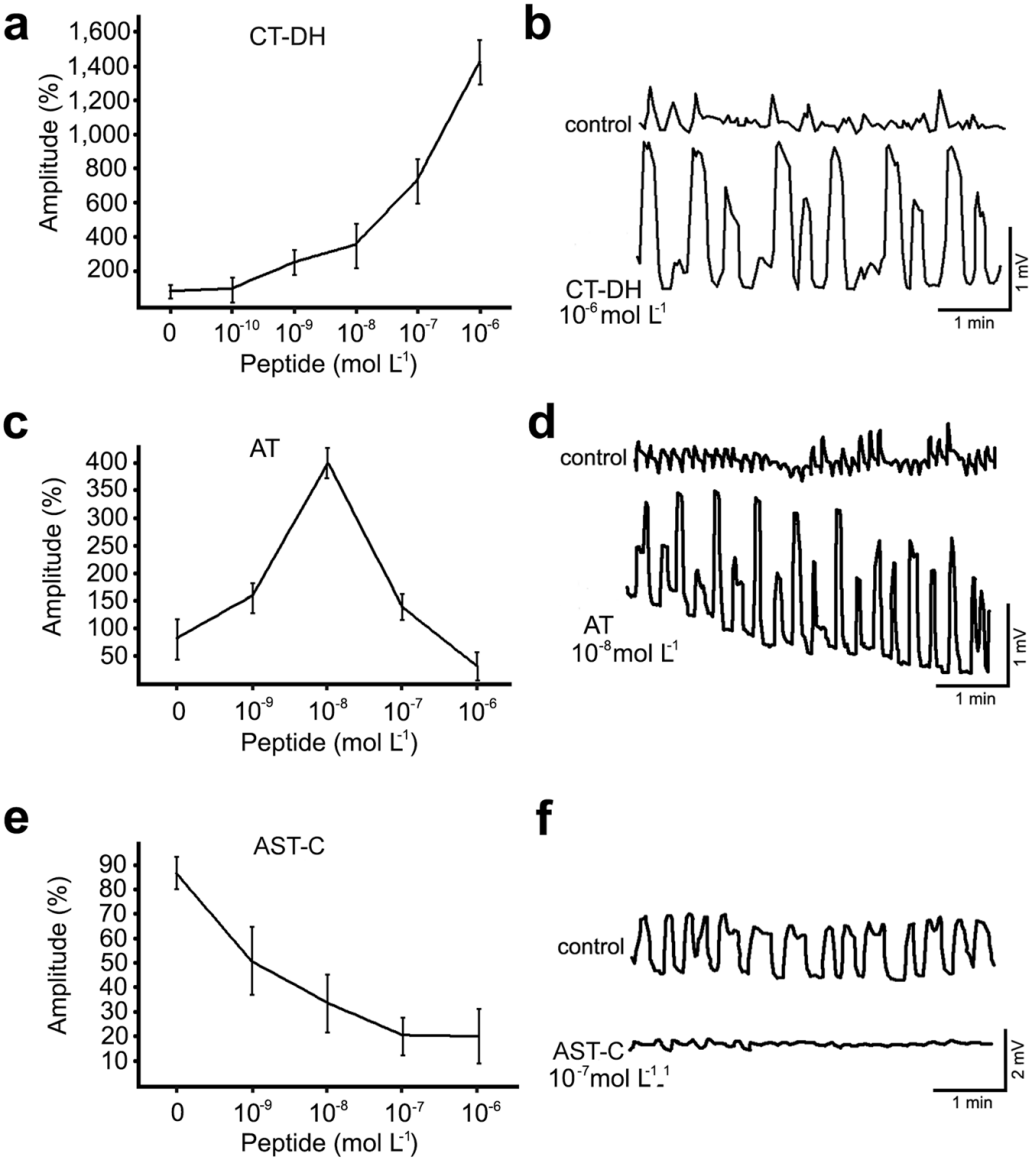
Modulation of frequency and amplitude of gonoduct contractions by different neuropeptides. (**a**) Dose-dependent stimulatory effect of CT-DH. (**b**) Gonoduct activity prior and after application of 10^−6^ mol L^−1^ CT-DH. (**c**) Dose-response curve of AT showing the best stimulatory effect at concentration 10^−8^ mol L^−1^. (**d**) Stimulation of spontaneous contractions by application of 10 nM AT. (**e**) Dose-response of AST-C showed myoinhibitory effect with the maximum at 10^−7^ mol L^−1^. (**f**) Traces showing inhibition of contractions after application of 10^−7^ mol L^−1^ AST-C. Data points in (**a,c,e**) are mean ± s.e.m. of 3–5 replicates. Note that differences in the amplitude of control samples (**b,d,f**) reflect individual variability, however stimulatory or inhibitory effects of different neuropeptides are very apparent.

Using a combination of ISH with MIP probe and immunostaining with MIP antibody we identified about 30 neurons in the larval TAG (Fig. 4a,b). Strong expression was observed in a pair of lateral interneurons 704 in the AN7 (IN704)^18^. Four large proctodeal medial neurons (PM8) and a pair of lateral neurons with axonal projection into the ipsilateral ventral nerve 8 (VL8) were detected in the AN8, while proctodeal lateral and medial neurons (PL9 and PM9) were the most prominent in the AN9. PM8, VL8 and PL9 also show FMRFamide-like-IR (Fig. 4b). In pharate pupae the number of MIP-positive neurons considerably increased up to 150. The expression pattern was very similar in both sexes and included larval prominent cells plus ~130 small neurons (6–10 μm) dispersed throughout the TAG (Fig. 4c,d). Relatively weak MIP expression appeared in MAN9 after pupation, but gradually increased in pharate adults in males (Fig. 4e). Both pharate males and females showed apparent MIP expression in IN704 and PM9, while most neurons detected in larvae and pupae disappeared or were only weakly stained (Fig. 4e,f). Immunohistochemical detection of MIP clearly revealed ∼20 somata of MAN9 (Fig. 4g) and 4–6 somata of PM9-like neurons in females (Fig. 4h).

### Innervation of reproductive tract by MAN9

The reproductive system of *B. mori* is innervated by terminal nerve branches from the TAG. The male-specific branch of the terminal nerve innervates the upper ejaculatory duct and accessory glands as described in *M. sexta*^4^. Using immunohistochemistry with antibodies to AT, AST-C, CT-DH and MIP we traced axonal projections of MAN9 neurons via this branch of terminal nerve to male gonads. This innervation forms a dense network of axonal branches with varicosities on surface of the accessory glands (Fig. 5a,b), basal part of vasa deferentia (Fig. 5c), seminal vesicles (Fig. 5d) and the ejaculatory duct (Fig. 5e). Origin of this peptidergic innervation could be traced to the terminal nerve that contains ~20 immunoreactive axons (Fig. 5f). Additional AT-, MIP- and *Bombyx* myosupressin (BMS)-immunoreactive nerve fibers containing varicosities on surface of the terminal nerves originate from some unknown central neurons (Fig. 5f; for MIP-IR see Fig. 3d′,e′).

### Effects of neuropeptides produced by MAN9 on the male genital tract

The male gonoducts exhibit spontaneous myoactivity *in vitro*. Each part of the reproductive tract shows contractions, but the most vigorous movements were observed in the boundary between seminal vesicles and ejaculatory duct. We observed two types of motion: continual peristaltic waves and rapid twitching of whole organs. CT-DH elicited the most remarkable increase in amplitude of contractions (Fig. 6a,b). The strongest effect (up to 20-fold stimulation) was observed after application of 10^−6^ mol L^−1^ CT-DH (Fig. 6a). The effect was exponential and neither declining nor plateau phase was observed using higher doses. Frequency of contractions was individually variable (8–22 contractions per 10 min; in average 16 per 10 min) and did not depend on the concentration of CT-DH (Fig. 6b).

Application of AT elicited quite different stimulatory effects (Fig. 6c,d). Only 4–5-fold stimulation of amplitude was achieved at 10^−8^ mol L^−1^, but higher doses (10^−7^, 10^−6^) led to inhibition of contractions (Fig. 6c). At 10^−8^ mol L^−1^ frequency of contractions increased to 24–50 per 10 min (Fig. 6d). AST-C exhibited an inhibitory activity on male gonoducts. Maximal 4-fold inhibition was observed at concentrations 10^−7^ mol L^−1^ and 10^−6^ mol L^−1^ (Fig. 6e,f).

**Figure 7:**
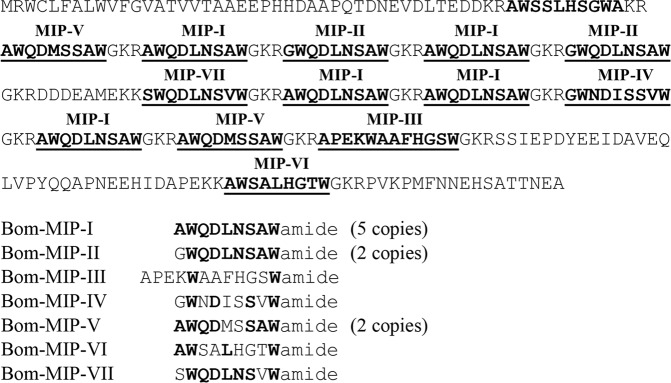
Amino acid sequence of MIP prepropeptide containing multiple copies of MIP-I-VII (bold, underlined) and alignment of MIP-I-VII showing amino acid identity in bold.

**Figure 8:**
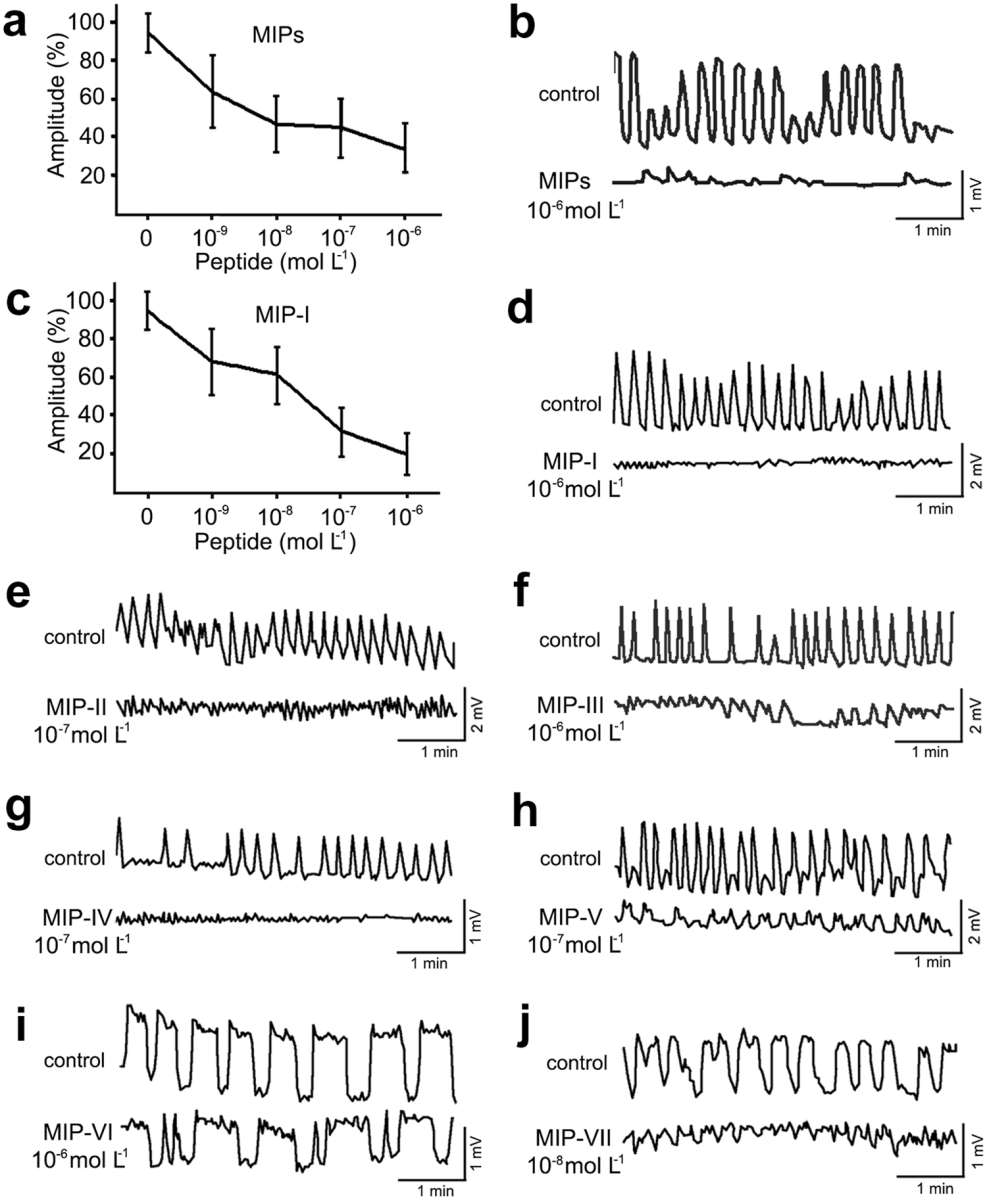
MIP-induced inhibition of spontaneous contractions of male gonoducts. (**a**) Dose-response curve showing the dose-dependent effect of MIP mixture. Concentration of each MIP corresponds to stoichiometry of peptides encoded by the precursor. The maximal inhibition was observed at 10^−6^ mol L^−1^. (**b**) Traces showing spontaneous muscle activity prior and after aplication 10^−6^ mol L^−1^ of this mixture. (**c,d**) Dose-response curve and traces showing inhibition of contractions after application of 10^−6^ mol L^−1^ MIP-I. (**e–j**) Other MIP peptides also suppressed spontaneous contractions. Traces showing spontaneous muscle activity prior and after application of 10^−7^ mol L^−1^ MIP-II (**e**), 10^−6^ mol L^−1^ MIP-III (**f**), 10^−7^ mol L^−1^ MIP-IV (**g**), 10^−7^ mol L^−1^ MIP-V (**h**), 10^−6^ mol L^−1^ MIP-VI (**i**) and 10^−8^ mol L^−1^ MIP-VII (**j**). Data points in (**a,c**) are mean ± s.e.m. of 3–5 replicates.

A mixture of MIPs was prepared from seven related peptides and their concentration reflects stoichiometry of each copy encoded by the propeptide precursor (Fig. 7). The dose-response curve shows an inhibitory effect of MIP mixture with 34% threshold inhibition at 10^−6^ mol L^−1^ concentration (Fig. 8a,b). The effect of each isoform was also examined separately (Fig. 8c–j). MIP-I showed the strongest effect with 5-fold inhibition at 10^−6^ mol L^−1^ (Fig. 8c,d). Most peptides elicited similar inhibitory effect at concentrations 10^−8^–10^−6^ mol L^−1^ (Fig. 8e–h,j), whereas weak inhibitory effect of MIP-VI was observed at 10^−6^ mol L^−1^ concentration (Fig. 8i).

### Expression of neuropeptide receptors in the male genital tract

**Figure 9:**
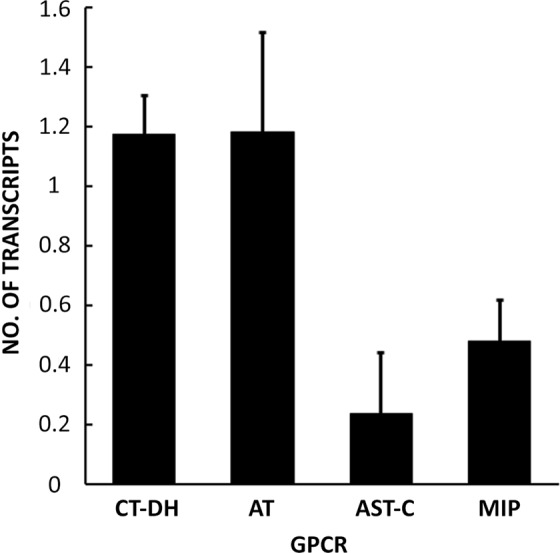
Expression analysis of neuropeptide receptors in the male reproductive organs. A mixture of the accessory glands, seminal vesicles and ejaculatory ducts of virgin males was analyzed. The vertical bars show numbers of transcripts per Rp49 and RpL3 RNA. Data are presented as a mean ± s.e.m. of 3 replicates.

Since neuropeptides produced by MAN9 modulated myoactivity of gonoducts *in vitro*, we next examined presence of their receptors in reproductive organs. These G-protein coupled receptors have been previously characterized^18–20^. Quantitative RT-PCR revealed that receptors for CT-DH (BNGR-B1), AT (BNGR-A16), AST-C (BNGR-A1) and MIP (sex peptide receptor) are all expressed in reproductive tissues innervated by MAN9. In the tissues of virgin male mRNAs of receptors for myostimulatory neuropeptides CT-DH and AT were 3–4-fold more abundant than receptors for inhibitory peptides AST-C and MIPs (Fig. 9).

## Discussion

In this paper we used immunohistochemistry, ISH and electrophysiology to determine differentiation and possible function of sex-specific cluster of neurons (MAN9) that innervate the male accessory glands and gonoducts in *B. mori*. This male-specific neuronal cluster has been first identified in *M. sexta* by cobalt backfilling and named “imaginal midline neurons of the abdominal segment 9” (A9 IMN)^4,5^. These authors described the appearance of peptide production in A9 IMN in pharate pupae that persisted to the adult stage using antibodies to small cardioactive peptide B (SCP-B) and FMRFamide^5,6^. Likewise, our ISH and immunohistochemical staining revealed that expression of neuropeptide transcripts and mature neuropeptides in MAN9 was initiated in pharate pupae and lasted to the adult stage of *B. mori*. These data indicate that MAN9 in *B. mori* are homologs of the posterior midline neurons described in the TAG of *M. sexta*. Peptidergic nature of this cluster was first revealed with antibodies to FMRFamide and SCP-B^5,6^. In a search for a possible FMRFamide-related peptide (FRP) produced by MAN9, we performed ISH with single stranded DNA probes specific for precursors encoding all known FRPs (BRF, BMS, NPY, NPF, sNPF, sulfakinin and RYamide). These probes showed strong and specific reactions in different neurons of the CNS, although none of them reacted in MAN9^8,15^. Based on these data, we concluded that FMRFamide antibody probably cross-reacted with RFamide C-terminus of MasATL-III. Indeed, preabsorption of our antibody to FMRFamide (diluted 1:3,000) with this peptide (10^−6^ mol L^−1^) abolished all immunoreactivity.

Our ISH, followed by immunohistochemical staining, clearly indicates that MAN9 express various neuropeptides (CT, AT, AST-C, MIPs) derived from four different precursors. Expression of three alternatively spliced transcripts encoding AT and AT-related peptides (ATLI, II, III) has been recently detected in MAN9 of *B. mori*^7^. Using AT antibodies or ISH with RNA probes these male-specific midline neurons have been previously described in other moths *M. sexta*, *Pseudaletia unipunctata* and *Heliothis virescens*^21–23^. This system is apparently not restricted to moths because a very similar sex-specific cluster of neurons innervating reproductive organs has been found in the cockroaches *Periplaneta americana* and *Leucophaea maderae*^24,25^. Moreover, AT-like myotropin was identified in extracts of the male accessory glands of the locust *Locusta migratoria*^26^. We assume that this peptide was isolated from innervation of the accessory glands. CT-DH is another peptide that may play an important role in reproduction, as indicated by a very strong expression of a receptor isoform CT-DH-R1-A in testes of the blood feeding bug *Rhodnius prolixus*^27^. In this species, proctolin-IR, allatostatin A-IR and FMRF-IR were also observed in innervation of reproductive organs^28–30^.

Our immunohistochemical staining showed that MAN9 innervate the accessory glands, seminal vesicles, basal part of the vasa deferentia and the upper ejaculatory duct suggesting involvement of this male-specific neuronal cluster in reproduction. Different neuropeptides produced by MAN9 either stimulated or inhibited spontaneous contractions of the accessory glands and gonoducts *in vitro* suggesting their role in male ejaculation during mating. *In vivo* action of MAN9 peptides on the gonoducts and accessory glands was corroborated by our expression analysis of receptors for AT, CT-DH, AST-C and MIPs. As expected, all these receptors were expressed in reproductive organs innervated by MAN9. Why so many excitatory and inhibitory peptides are coexpressed in MAN9 to control activity of the male reproductive system? Our electrophysiology data indicate a specific role of each neuropeptide that is present in innervation of the male accessory glands; CT-DH considerably increased amplitude, while AT increased frequency of contractions. The corelease of remaining inhibitory peptides (AST-C and MIPs) may enhance function of AT and CT-DH by inhibition of other neuronal or humoral inputs to the accessory glands. These inputs may be perceived through additional neuropeptides released from different neurons in the CNS. For example, a neuropeptide corazonin produced by four male-specific abdominal neurons controls copulation duration and sperm transfer via action on serotonergic neuronal cluster innervating the accessory glands of the fruitfly *Drosophila melanogaster*^31^. Similar abdominal neurons producing corazonin are present in various unrelated insects^32^, so it is plausible to speculate that this neural pathway may be involved in reproductive behaviors of different insect groups. Additional neuropeptides SIFamide, neuropeptide F and natalisin produced by the brain of many insects modulate sexual behaviors and fecundity in *D. melanogaster* and the beetle *Tribolium castaneum*^33–35^.

In conclusion we determined and described neuropeptide expression patterns in MAN9 during metamorphosis using ISH and immunohistochemistry, while electrophysiology data *in vitro* revealed possible functions of individual neuropeptides that coordinate activity of the male reproductive organs. Spatial and temporal analysis of receptor expression in different parts of gonads may provide clues for specific action of these different neuropeptides. Further functional analyses *in vivo* and *in vitro* using transgenic animals and RNAi approaches are essential to unravel complex relationships between neuropeptides from MAN9 and other neural pathways controlling sexual behaviors^36^.

## Materials and Methods

### Experimental animals

Polyvoltine hybrid of *B. mori* N4 strain was used for all experiments. Larvae were fed on mulberry leaves or standard artificial diet (Mulberry Farms, Fallbrook, CA, USA), at 25 °C under a 16:8 h light:dark photoperiod. For analysis of spatial and temporal expression of peptides, the following stages were used: feeding and pharate 4^th^ and 5^th^ instar larvae (20–12 h prior to ecdysis); wandering larvae, stop spinning larvae (larvae with reduced mobility at the end of the spinning phase), pharate pupae, pupae on day 0 and 2 of pupal ecdysis; pharate and freshly eclosed adults. Freshly eclosed virgin males were used in contraction assay.

### Molecular cloning

The brains and ventral nerve cords of the 5th instar larvae were dissected, immediately frozen on dry ice and stored at −80 °C until use. Total RNA was prepared using Trizol Reagent (Invitrogen, Carlsbad, CA, USA) and the double stranded cDNA for the PCR cloning was generated using SMART cDNA synthesis kit (BD Biosciences, CA, USA). The previously identified gene transcripts for studied peptides served for design of PCR primers using Primer3 software^37^. DreamTaq DNA Polymerase (Thermo Fisher Scientific, MA, USA) and primers listed in Table 1 were used in PCR. The peptide specific cDNA amplicons were purified with a QIAquick PCR purification kit (QIAGEN, Hilden, Germany) and custom sequenced.

**Table 1:** Primers used for production of hybridization probes and qRT-PCR.

Gene	Name	Primers (L - left primer; R – right primer)	Method
AST-C	NM_001130884	L: GCGTTTAGGCGAAGATGAAA R: CGCGAACACACTGATCAAAT	ISH
AT	AY970687	L: AGCAGGCAGTCTCACGAGTT R: CGGGTTGTTGAGAACCTCAT	ISH
CT-DH	NM_001130907	L: GCTTTCGATCTTGGTCTTGG R: TCAACGCAGAGTACTTTTTTTT	ISH
MIP	AB073553	L: GGTGTTTATTCGCGCTGTG R: TAGGAGCCTGCTGGTAAGGA	ISH
AST-C receptor	NM_001134264.1	L: TACTGCAACGCTTCAACCAC R: CATCAGCATCCAGTCATCTTG	qPCR
AT receptor	NM_001134242.1	L: TTCCCGCTGAAGTTCAAGTC R: GAAGCGCAGAGGAACAAATC	qPCR
CT-DH receptor	AB330457.1	L: CGCGACCTTCTCCTCTGC 1 R: ACGAACTCGGGACAAGAACC	qPCR
MIP/SP receptor	NM_001114874.1	L: TTCTGCGCATCAAACAACTC R: GATAGCACCACCACGATCAG	qPCR
RpL3	AB024901.1	L: AGCACCCCCTCATGGGTCTA R: TGCGTCCAAGCTCATCCTGC	qPCR
Rp49	NM_001098282.1	L: CAGGCGGTTCAAGGGTCAATAC R: TGCTGGGCTCTTTCCACGA	qPCR

### *In situ* hybridization

Wholemount ISH was performed essentially as described by Kim *et al*.^38^ and Roller *et al*.^8^. Briefly, digoxigenin-labeled single stranded DNA probes were synthesized by asymmetric PCR using the gene transcript specific right primers (Table 1). The purified amplicons (see Molecular cloning) were used as templates for the probe synthesis. Tissues from 6 to 10 individuals were examined for each studied stage and each hybridization probe. Negative controls were performed using the specific sense probes and positive controls using the previously characterized probes to neuropeptide gene transcripts^8,39^. The tissues with hybridized probes were stained under a binocular microscope and thereafter either mounted in glycerol or subjected to the immunohistochemical procedure. The preparations were observed and photographed using a fluorescent microscope Nikon Eclipse 600 with Nomarski DIC optics and attached Nikon Coolpix 990 camera (Nikon, Tokyo, Japan).

### Immunohistochemistry

The wholemount immunohistochemistry was conducted as previously described^14,15^. Detection of two peptide antigens in the same sample was performed using a mixture of primary antibodies generated in different animals (Table 2). Specificity of the used antibodies was tested by liquid-phase pre-absorption of the working dilution with 100 nM of the respective synthetic antigen (24 hours at 4 °C), and by comparison of staining pattern with the cellular distribution of the respective gene transcripts using ISH. Bound antibodies were visualized following incubation with a mixture of Alexa Fluor 488-labeled donkey anti-rabbit IgG and Alexa Fluor 594-labeled donkey anti-mouse (Jackson Immunoresearch Europe, Suffolk, UK; multiple labeling grade secondary antibodies; all diluted to 1:1,000). The preparations were observed and scanned by TCS SPE (Leica, Germany) confocal system using 488 and 532 nm lasers for excitation. Alignment of identical images from light and fluorescent microscopy and layout of composite images were performed using Adobe Photoshop 7.0 (Adobe Systems, San Jose, CA).

**Table 2:** Antibodies used in this study.

Antibody to	Host, dilution	Reference
AT	Rb 1:2,000	^43^
AST-C	Rb 1:1,000	^43^
CT-DH	Rb 1:2,000	^44^
	Mp 1:1,000	^39^
FMRFamide	Rb 1:3,000	^45^
MIP	Mm 1:1,000	^38^
myosupressin (BMS)	Mm 1:1,000	^46^
Orcokinin (ORC)	Mp 1:4,000	^39^

Mm, mouse monoclonal; Mp, mouse polyclonal; Rb, rabbit polyclonal.

### Quantitative RT-PCR

The accessory glands, seminal vesicles and ejaculatory ducts of virgin males were dissected, and stored in RNAlater stabilization reagent (QIAGEN) at +4 °C until use. A mixture of organs from three individuals were pooled for RNA isolation. Total RNA was prepared using RNeasy Protect Mini Kit (QIAGEN) and the single stranded cDNA was generated using oligo(dT)_18_ primers and Maxima H Minus First Strand cDNA Synthesis Kit (ThermoFisher Scientific, MA, USA). Transcripts were quantified on a real time PCR machine CFX96 (Bio-rad, Hercules, CA, USA) using cycling program according to the manufacturer instructions. All primers were employed at hybridization temperature 60 °C and their sequences are shown in Table 1. Each primer was designed to match distinct genomic exon to avoid false positive amplification from gDNA. PCR was performed using XceedqPCR SG Mix (2x) Lo-ROX kit (Institute of Applied Biotechnologies, Praha, Czech Republic). Transcript levels of analysed receptors were measured in three technical replicates and normalized to the levels of reference genes RpL3 and Rp49 in the same samples. Three biological replicates were used for each quantitative analysis.

### Neuropeptides

Synthetic BomCT-DH31 was obtained from Dr. D. A. Schooley (Univ. of Nevada, USA), allatotropin was purchased from Clonestar peptide services (Brno, Czech Republic), MIPs were produced as described^17^ and AST-C was obtained from Dr. N. Audsley. All peptides were dissolved in distilled water and stored as aliquots (10^−3^ mol L^−1^) at −20 °C until used in contraction assay.

### Contraction assay

Contractions of a region where the seminal vesicle joins the ejaculatory duct were analyzed. The whole male reproductive tract with attached gonads and accessory glands were dissected in Weevers saline^40^ and secured with minutien pins in a Petri dish coated with Sylgard (Dow Corning Corporation, Midland, MI, USA). Thin metal wire was used to connect a distal part of the seminal vesicle to the strain-gauge transducer^41^. The signal was amplified using the four channel tensometric unit M-1000 (Mikrotechna Co., Praha). Contractions were recorded and analyzed using WINDAQ 2.59 software (DATAQ Instruments Inc., OH, USA). All preparations were maintained in 300 μl of Weevers saline. Initially, the spontaneous contractions were recorded for 10 min and then a peptide or a mixture of peptides diluted in 30 μl saline were applied to the preparation. Peptide-induced changes were recorded for 30–40 min and the average amplitude and frequency of contractions were counted from 2–3 different time points of recordings. Values elicited by peptides were expressed as a percentage of the average amplitude of the control spontaneous contractions in that measurement. Three to five repeats were recorded for each peptide concentration. The results in the dose-response charts are presented as means ± standard error of the mean (s.e.m.).

## Data Availability

The data generated and analyzed in the current study are available by the corresponding author.
